# Improved Electrochemical Performance of 0.5Li_2_MnO_3_·0.5LiNi_0.5_Mn_0.5_O_2_ Cathode Materials for Lithium Ion Batteries Synthesized by Ionic-Liquid-Assisted Hydrothermal Method

**DOI:** 10.3389/fchem.2020.00729

**Published:** 2020-11-23

**Authors:** Yanhong Xiang, Youliang Jiang, Saiqiu Liu, Jianhua Wu, Zhixiong Liu, Ling Zhu, Lizhi Xiong, Zeqiang He, Xianwen Wu

**Affiliations:** ^1^School of Physics and Mechanical and Electrical Engineering, Jishou University, Jishou, China; ^2^College of Biology and Environmental Sciences, Jishou University, Jishou, China; ^3^School of Chemistry and Chemical Engineering, Jishou University, Jishou, China

**Keywords:** lithium ion battery, Li-rich Mn based, cathode materials, hydrothermal, ionic liquid

## Abstract

Well-dispersed Li-rich Mn-based 0.5Li_2_MnO_3_·0.5LiNi0.5Mn_0.5_O_2_ nanoparticles with diameter ranging from 50 to 100 nm are synthesized by a hydrothermal method in the presence of N-hexyl pyridinium tetrafluoroborate ionic liquid ([HPy][BF4]). The microstructures and electrochemical performance of the prepared cathode materials are characterized by X-ray diffraction (XRD), scanning electron microscopy (SEM), transmission electron microscopy (TEM), and electrochemical measurements. The XRD results show that the sample prepared by ionic-liquid-assisted hydrothermal method exhibits a typical Li-rich Mn-based pure phase and lower cation mixing. SEM and TEM images indicate that the extent of particle agglomeration of the ionic-liquid-assisted sample is lower compared to the traditional hydrothermal sample. Electrochemical test results indicate that the materials synthesized by ionic-liquid-assisted hydrothermal method exhibit better rate capability and cyclability. Besides, electrochemical impedance spectroscopy (EIS) results suggest that the charge transfer resistance of 0.5Li_2_MnO_3_· 0.5LiNi0.5Mn_0.5_O_2_ synthesized by ionic-liquid-assisted hydrothermal method is much lower, which enhances the reaction kinetics.

## Introduction

Rechargeable lithium-ion batteries (LIBs) have conquered the electronics field due to its many advantages such as high energy and power density, largest output voltage, long cyclic life, and environmental friendliness, compared to other rechargeable batteries (Zhou et al., 2016; Nie et al., 2020; Shi et al., 2020; Tang et al., 2020). However, owing to the rapid development of electric vehicles, portable electronics, and stationary energy storage devices, new advances in performances/safety/costs are needed (Li et al., 2017; Lu et al., 2017; Yang et al., 2019; Guo et al., 2020; Zheng et al., 2020a). Therefore, the alternative cathode materials with high specific capacities have been extensively explored. Compared with the conventional commercial cathode materials, layered lithium-rich manganese-based cathode materials, *x*Li_2_MnO_3_·(1 – *x*)LiMO_2_ (M = Mn, Ni, Co, etc.), which consist of two components of α-NaFeO_2_-structured LiMO_2_ (R-3m symmetry) phase and monoclinic Li_2_MnO_3_ (C2/m symmetry) phase, have attracted extensive interest due to their low price and higher discharge capacity of more than 250 mA h g^−1^ (Lin et al., 2008; Yan et al., 2014; Nayak et al., 2018; Yang et al., 2018; Xiang et al., 2019; Gao et al., 2020; Jiang et al., 2020; Zhao et al., 2020). However, several drawbacks, including intrinsic poor capability, poor cycling stability, and voltage fading, hindered its practical applications (Song et al., 2016; Xiang et al., 2017; Hu et al., 2019; Zhang et al., 2019; Sigel et al., 2020).

To solve these problems, plenty of efforts have been made to realize electrochemical performance improvement, including synthesizing strategies (Hua et al., 2019; Redel et al., 2019), elemental doping (Yu et al., 2019; Zheng et al., 2020b), and surface modification (Liu et al., 2019; Peng et al., 2020; Zhong et al., 2020). Many research groups have recently adopted different synthetic methods to improve the electrochemical performance of Li-rich Mn-based layered cathode materials because of the optimization of suitable morphologies and sizes, such as coprecipitation method, sol–gel, and hydrothermal method (Pimenta et al., 2017). Among these methods, the hydrothermal method is promising since it has many advantages over other methods, such as homogeneous mixing at the atomic or molecular level, high purity, and small particle size. Usually, lowering the particle size can considerably meet the improvement of the rate capability (Deng et al., 2020). However, the hydrothermal method has several drawbacks such as uneven distribution and serious agglomeration.

Ionic liquids (ILs), which are room temperature molten salts, consisting of organic cations and inorganic anions, have been recently used to synthesize nanomaterials with desirable structures and morphologies due to their unique physical and chemical properties (Li et al., 2008). Therefore, we use the [HPy][BF_4_] ionic liquid as both solvent and template to enable the growth of 0.5Li_2_MnO_3_·0.5LiNi_0.5_Mn_0.5_O_2_ powders with controlled size and morphology, which are expected to improve the rate capability of this material. The microstructures and electrochemical performance of the prepared cathode materials are investigated.

## Experimental

### Materials Synthesis

**Figure 1:**
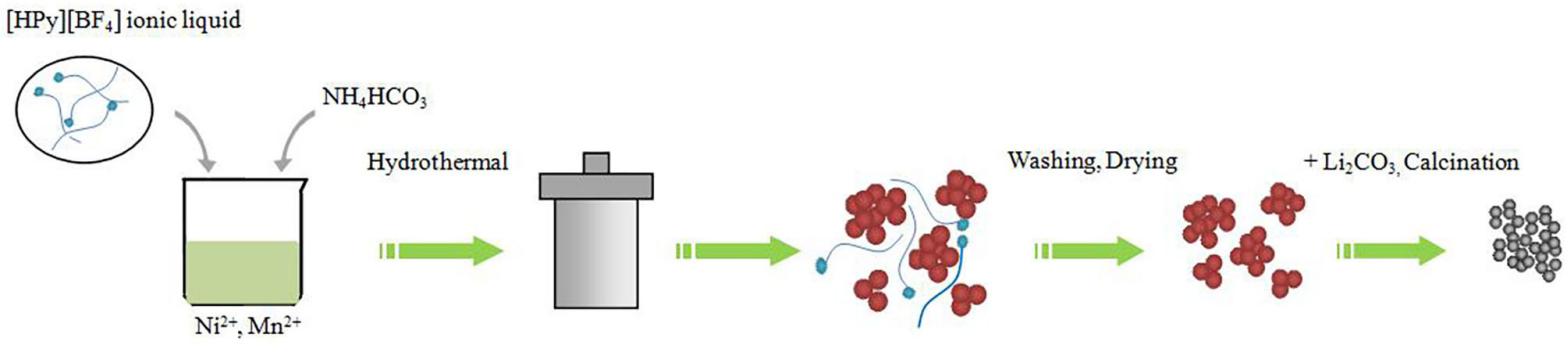
Illustration of the formation of 0.5Li_2_MnO_3_·0.5LiNi_0.5_Mn_0.5_O_2_ nanoparticles with ionic-liquid-assisted hydrothermal method.

All the raw materials were analytical reagent and used without further purification. 0.5Li_2_MnO_3_·0.5LiNi_0.5_Mn_0.5_O_2_ was prepared by ionic-liquid-assisted hydrothermal method as follows (Figure 1). A stoichiometric amount of Ni(CH_3_COO)_2_·4H_2_O and Mn(CH_3_COO)_2_·4H_2_O together with 1 g of [HPy][BF_4_] ionic liquid was dissolved in deionized water. The NH_4_HCO_3_ aqueous solution, which was used as the precipitation reagent, was pumped into the obtained mixed solution drop by drop under magnetic stirring. Finally, the resultant mixture was sealed into a 100-ml Teflon-lined stainless steel autoclave and maintained at 180°C for 12 h. After being cooled down to ambient temperature, the suspension was filtered, and the precipitated powders were washed and then dried at 105°C for 5 h. The obtained carbonate precursors were thoroughly mixed with a 3-wt% excess of a stoichiometric amount of Li_2_CO_3_ to offset the possible evaporative of lithium occurring at high temperature and then calcined at 700°C for 10 h in air. At the same time, the sample without adding ionic liquid was synthesized for comparison.

### Characterization

Thermogravimetric (TG) and differential scanning calorimetry (DSC) analysis was tested by an SDTQ600 analyzer, at a rate of 10°C min^−1^ under air conditions in the temperature range of 25–900°C. The crystal nature of materials was identified by X-ray diffraction (XRD, Rigaku 2500, Japan) with Cu-*K*α radiation. The particle morphology, size, and distribution of the as-synthesized powders were observed by scanning electron microscopy (SEM, JEOL JSM-5600LV) and transmission electron microscopy (TEM, Tecnai G12).

### Electrochemical Test

The electrode slurry was prepared by mixing 0.5Li_2_MnO_3_·0.5LiNi_0.5_Mn_0.5_O_2_ power, carbon black, and polyvinylidene difluoride (PVDF) with a weight ratio of 8:1:1 in *N*-methyl-2-pyrrolidone (NMP). The slurry was cast onto an Al foil and then cut into a circular electrode after dried. The testing coin-type cells (CR2032) of Li | LiPF_6_ (EC/DEC/DMC = 1:1:1 by volume) | 0.5Li_2_MnO_3_·0.5LiNi_0.5_Mn_0.5_O_2_ were assembled in an argon-filled glove box. The cathode and anode electrodes were separated by Celgard 2400 films. The charge–discharge texts were carried out by using the LAND-CT2001A battery text system (Wuhan, China). The electrochemical impedance spectroscopy (EIS) was tested by an electrochemical workstation CHI660E over a frequency range of 100 kHz to 0.01 Hz.

## Results and Discussion

**Figure 2:**
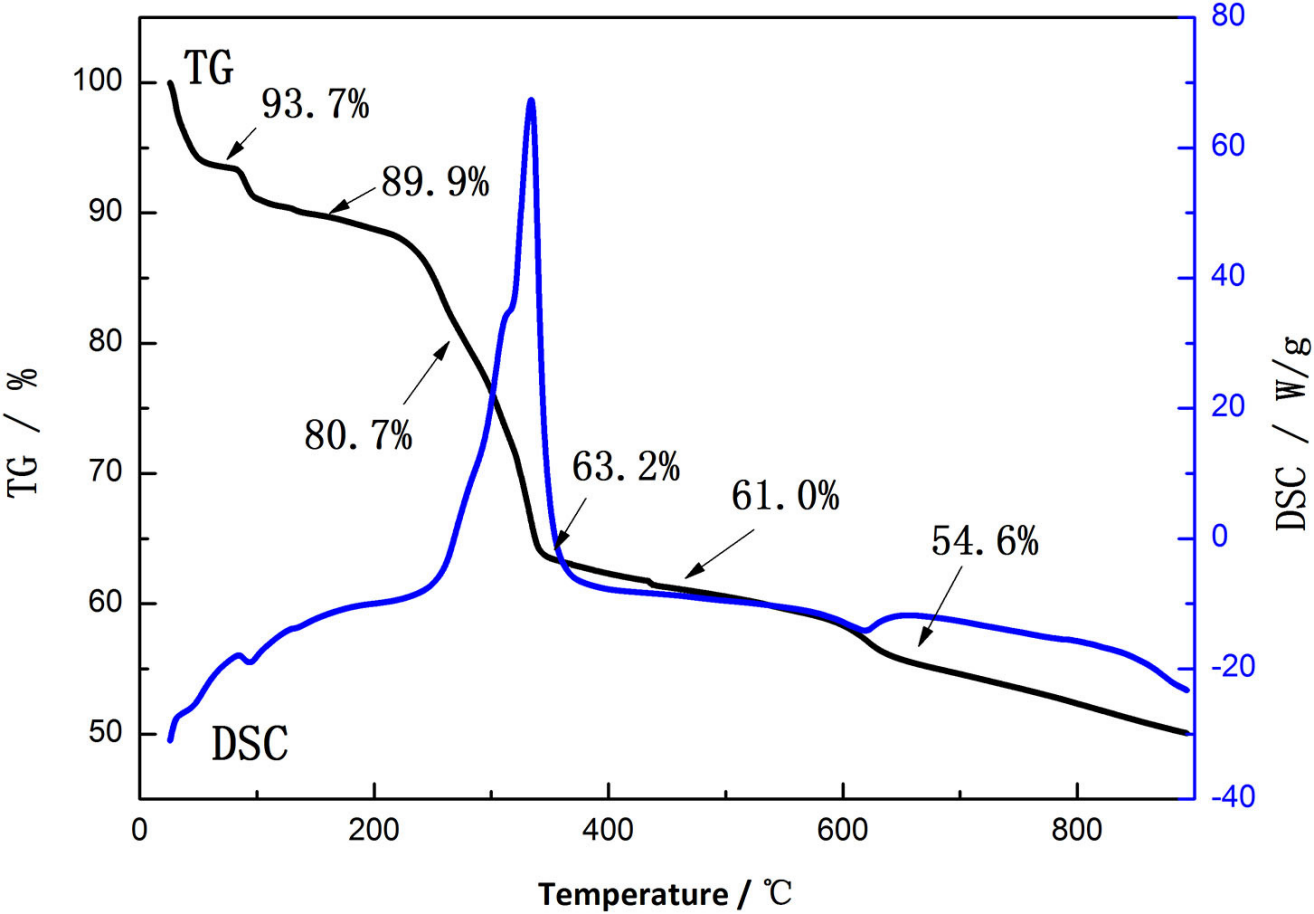
Thermogravimetric–differential scanning calorimetry (TG-DSC) curves of the mixture of Li_2_CO_3_ and the precursor synthesized by ionic-liquid-assisted hydrothermal method.

Figure 2 shows the TG-DSC patterns of the mixture of Li_2_CO_3_ and the precursor synthesized by ionic-liquid-assisted hydrothermal method. There are three weight losses that appear at 80–100°C, 220–350°C, and 600–640°C in the TG-DSC patterns, respectively, which correspond to the evaporation of free water, the decomposition of the mixture and a small amount of residual ionic liquids, and the formation of lattice oxides, respectively. Therefore, 700°C is chosen as the sintering temperature.

**Figure 3:**
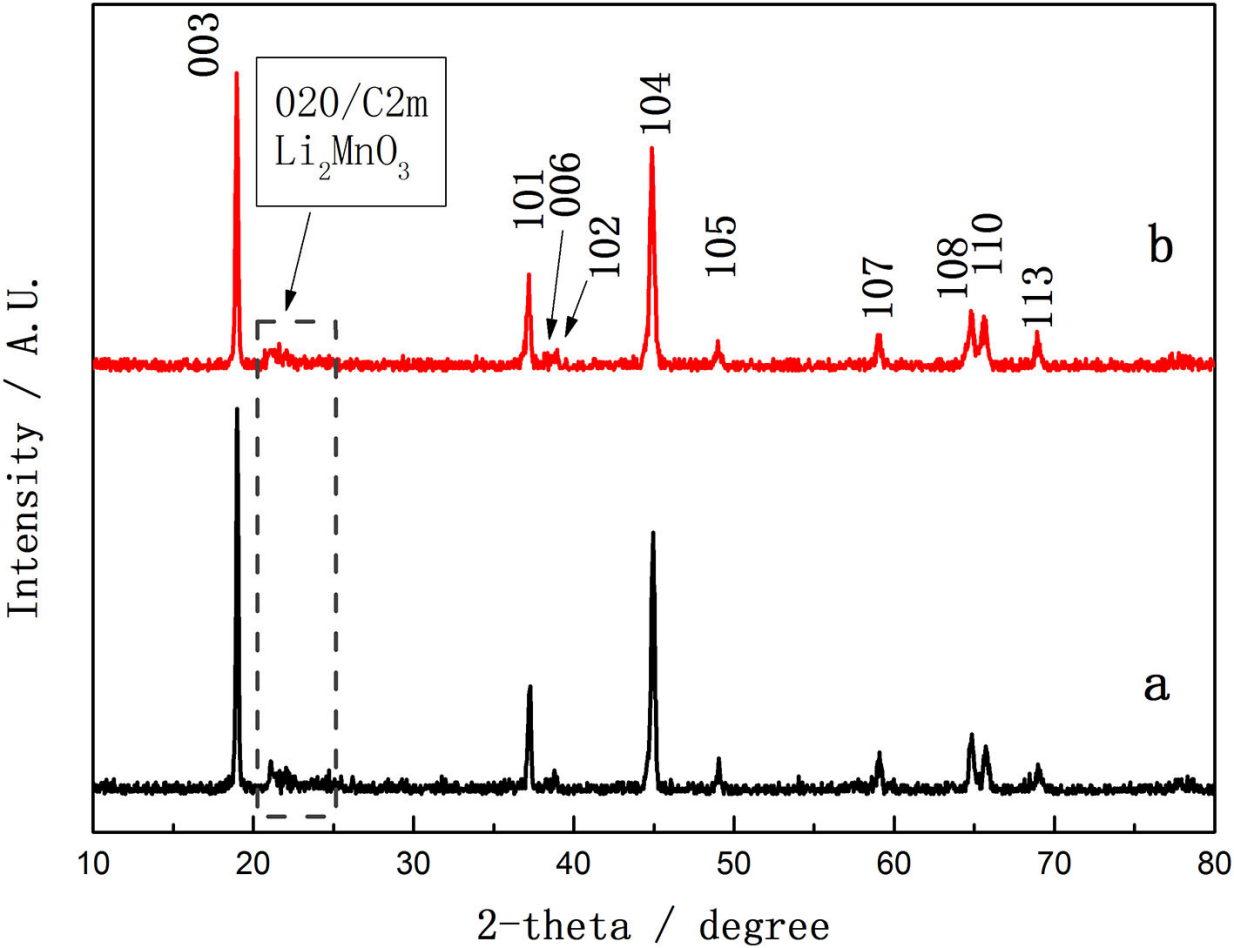
The X-ray diffraction (XRD) patterns of (a) TH-LMNO and (b) ILH-LMNO.

Figure 3 compares the XRD patterns of 0.5Li_2_MnO_3_·0.5LiNi_0.5_Mn_0.5_O_2_ precursor synthesized by the traditional hydrothermal method (TH-LMNO) and ionic-liquid-assisted hydrothermal method (ILH-LMNO). The crystal lattice of both samples can be approximated to the hexagonal α-NaFeO_2_-type structure (R-3m) according to the strong diffraction peaks, except for the low-intensity peaks at 20–25°, corresponding to a monoclinic Li_2_MnO_3_ phase (C2/m symmetry) (Thackeray et al., 2006, 2007; Johnson et al., 2008), is highlighted in Figure 3. The obvious split (006)/(102) and (108)/(110) peaks indicate that the synthesized powders have a good layered structure. No peaks of any impurity phase are detected in the XRD patterns of the ILH-LMNO sample, indicating that the ionic liquid has no effects on the crystal structure of the final materials. The ratio (*R*) of *I*_(003)_/*I*_(104)_ is used to determine the degree of cation mixing of layered structure. When the *R* value is >1.2, it is suggested that the degree of cation mixing is small and the materials have an excellent hexagonal layered structure. The *R* value of the ILH-LMNO sample is 1.61, larger than the value of the TH-LMNO sample (1.40), suggesting a low cation mixing and better crystalline structure, which implies excellent rate capability and cyclic performance.

**Figure 4:**
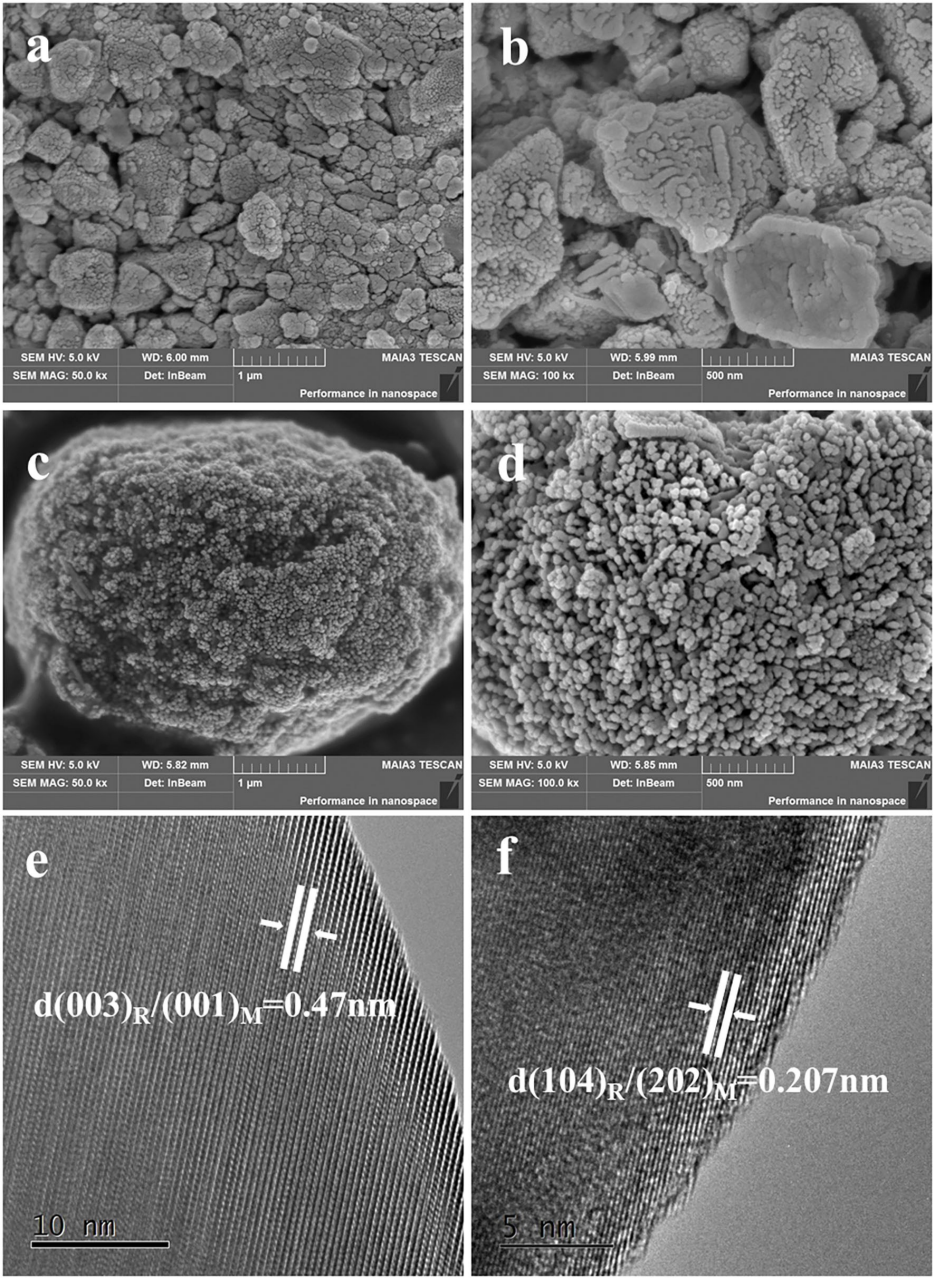
The scanning electron microscopy (SEM) images of **(a,b)** TH-LMNO and **(c,d)** ILH-LMNO. The transmission electron microscopy (TEM) images of **(e,f)** ILH-LMNO.

Scanning electron microscopy images of TH-LMNO and ILH-LMNO are examined and shown in Figure 4. Compared with the obvious agglomeration particles for the TH-LMNO sample, the primary particles of the ILH-LMNO sample are more uniform and dispersed. The molecules of [HPy][BF_4_] can be adsorbed on the surfaces of the particles during the formation process of precursor, preventing the particles aggregating mutually due to their relative large organic cations (C_11_H_18_N^+^), which act as barriers. Figure 1 shows schematic effect of [HPy][BF_4_] and formation process of 0.5Li_2_MnO_3_•0.5LiNi_0.5_Mn_0.5_O_2_. Due to the template function of the [HPy][BF_4_] ionic liquid, a large number of pores formed as well as smaller, more uniform particles obtained. The aggregates of the ILH-LMNO sample are arranged in chains, and the particles are distributed in a narrow range of 50–100 nm. In general, the small size of the nanoparticles will reduce the diffusion path of Li^+^ ions and electrons and thus enhance the rate capability of the materials (Wu et al., 2017; Zhao et al., 2018). The morphologies and microstructures of the ILH-LMNO sample were further clarified by high resolution TEM (HRTEM) characterization. The width between lattice fringes in Figure 4e is 0.47 nm, which correspond to the (003) plane of the LiNi_0.5_Mn_0.5_O_2_-layered structure (R-3m) or (001) plane of the Li_2_MnO_3_ superlattice structure (C-2m), while the width between lattice fringes in Figure 4f is 0.207 nm, corresponding to the (104) plane of the LiNi_0.5_Mn_0.5_O_2_-layered structure or (202) plane of the Li_2_MnO_3_ superlattice structure, demonstrating that the lithium-rich manganese-based 0.5Li_2_MnO_3_·0.5LiNi_0.5_Mn_0.5_O_2_ materials can be successfully prepared by the ionic-liquid-assisted hydrothermal method.

**Figure 5:**
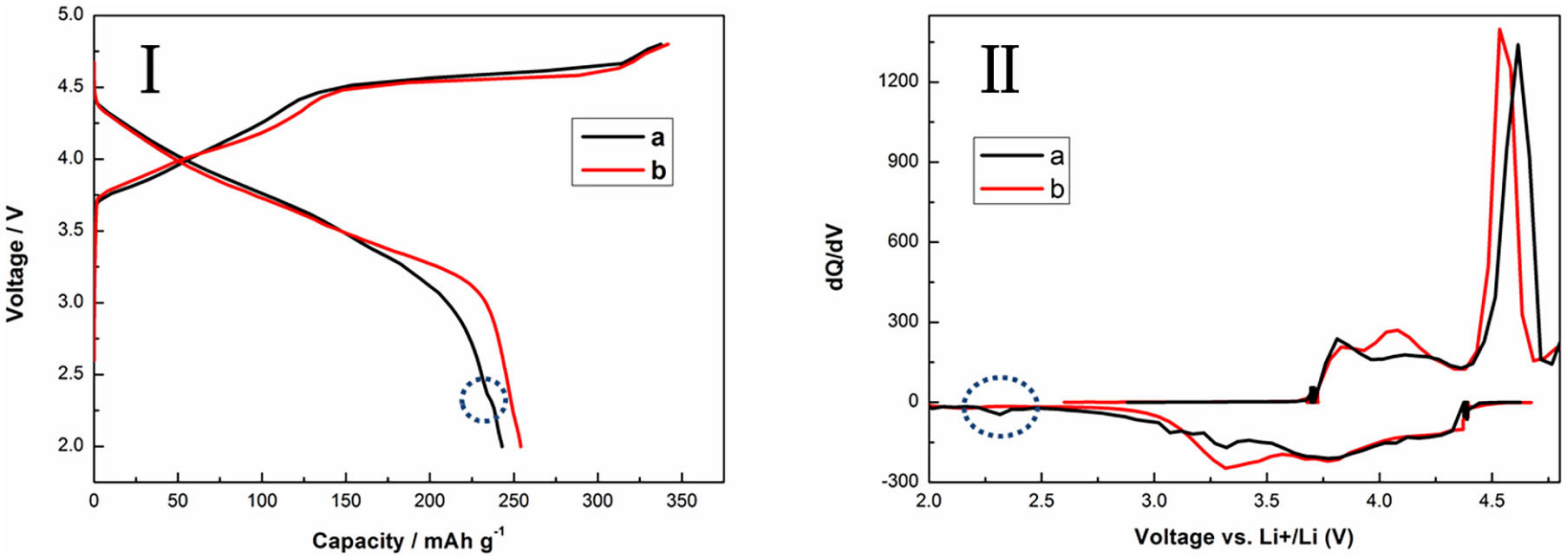
The initial charge/discharge curves (I) and the dQ/dV plots (II) of (a) TH-LMNO and (b) ILH-LMNO (4.8–2.0 V, 25 mA g^−1^).

Electrochemical evaluations of the TH-LMNO and ILH-LMNO electrodes are performed in lithium coin-type cells. Figure 5 shows the typical voltage profiles and the dQ/dV plots of the initial charge/discharge of TH-LMNO and ILH-LMNO at 0.05 C (25 mA g^−1^) between 2.0 and 4.8 V. As can be seen, all the charging profiles in the figure are composed of two regions, which correspond to the two prominent anodic peaks in the dQ/dV curves. The slope region below 4.5 V is due to the extraction of Li^+^ ions from the LiNi_0.5_Mn_0.5_O_2_ component with Ni oxidation from Ni^2+^ to Ni^4+^. The other plateau region above 4.5 V is attributed to the removal of Li_2_O from the layered Li_2_MnO_3_ component with “MnO_2_-like” activation, which results in not only the extraordinary capacity but also the larger initial irreversible capacity loss offered by Li-rich materials (Oishi et al., 2015; Assat and Tarascon, 2018). Both the cell with TH-LMNO and ILH-LMNO have three reduction prominent peaks during the initial discharge process at 3.2, 3.7, and 4.3 V, except an additional Li uptake peak at 2.3 V for the TH-LMNO sample, which can be assigned to a spinel phase (Riekehr et al., 2016). The cation distribution of the ILH-LMNO sample is uniform, and there has no heterogeneous production, so there is no peak at 2.3 V. The peaks at ~4.3 and ~3.7 V can be attributed to the Ni^4+^/^3+^/^2+^ reduction, and the peak around 3.2 V is associated with the Mn^4+^/^3+^ reduction (Johnson et al., 2008; Chen et al., 2015). ILH-LMNO electrode provided a significantly higher capacity (254.1 mA h g^−1^) together with superior first-cycle efficiency (74.4%) as compared to the TH-LMNO electrode (242.9 mA h g^−1^, 72.1%).

**Figure 6:**
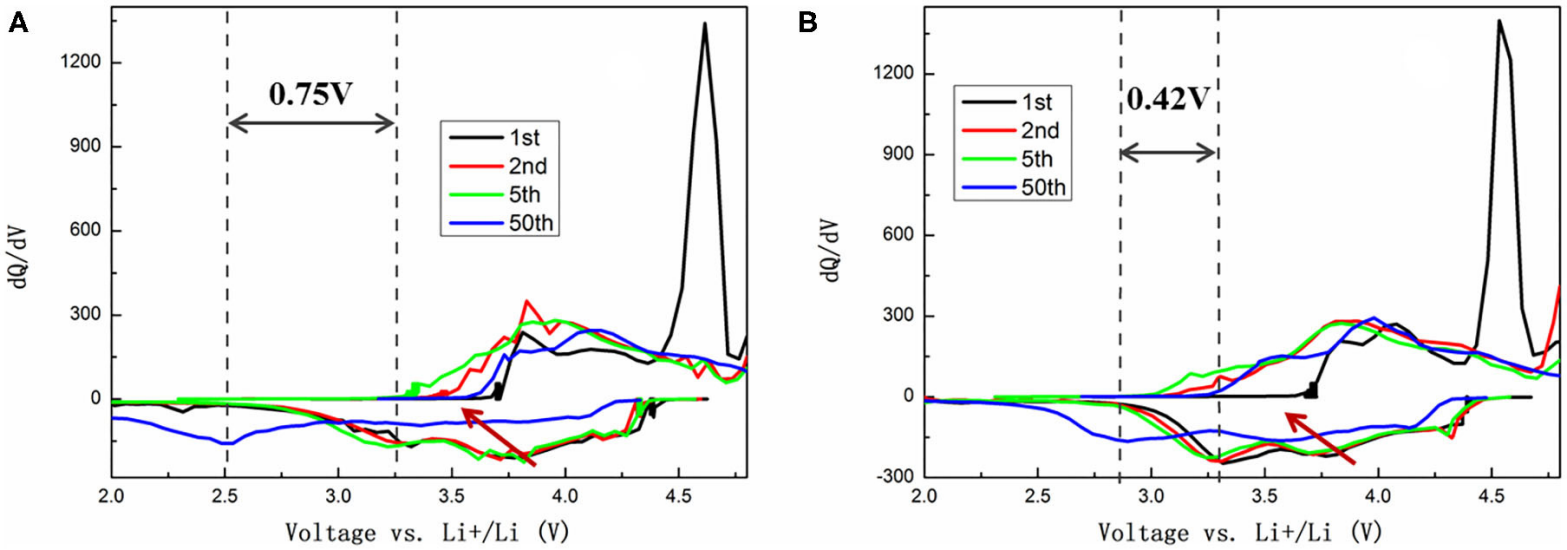
The dQ/dV plots of **(A)** TH-LMNO and **(B)** ILH-LMNO.

To further identify and understand the redox reactions between the first and the following cycles, the evolution of dQ/dV profiles corresponding to the 1st, 2nd, 5th, and 50th cycles of TH-LMNO and ILH-LMNO are provided in Figure 6. It is clearly found from the dQ/dV curves that the peak above 4.5 V in the initial cycle is irreversible and disappears in the subsequent cycles, demonstrating that the activation of Li_2_MnO_3_ component is irreversible. Meanwhile, all the reduction peaks shift continuously to a lower voltage upon cycles, which is attributed to the voltage decay due to layered-to-spinel phase transitions (Yu et al., 2019). It can be seen that the Ni^4+/3+/2+^ reduction peaks decrease more significantly upon cycles for the TH-LMNO sample compared with the ILH-LMNO sample. In addition, the reduction peak of Mn^4+/3+^ at ~3.2 V decreased 0.75 V after the 50th cycle for the TH-LMNO sample, while the reduction peak decreased only 0.42 V for the ILH-LMNO sample. All the above characteristics illustrate that the ILH-LMNO sample has better capacity and voltage retention, which is mainly due to the more uniform particles and better cation arrangement of the ILH-LMNO sample.

**Figure 7:**
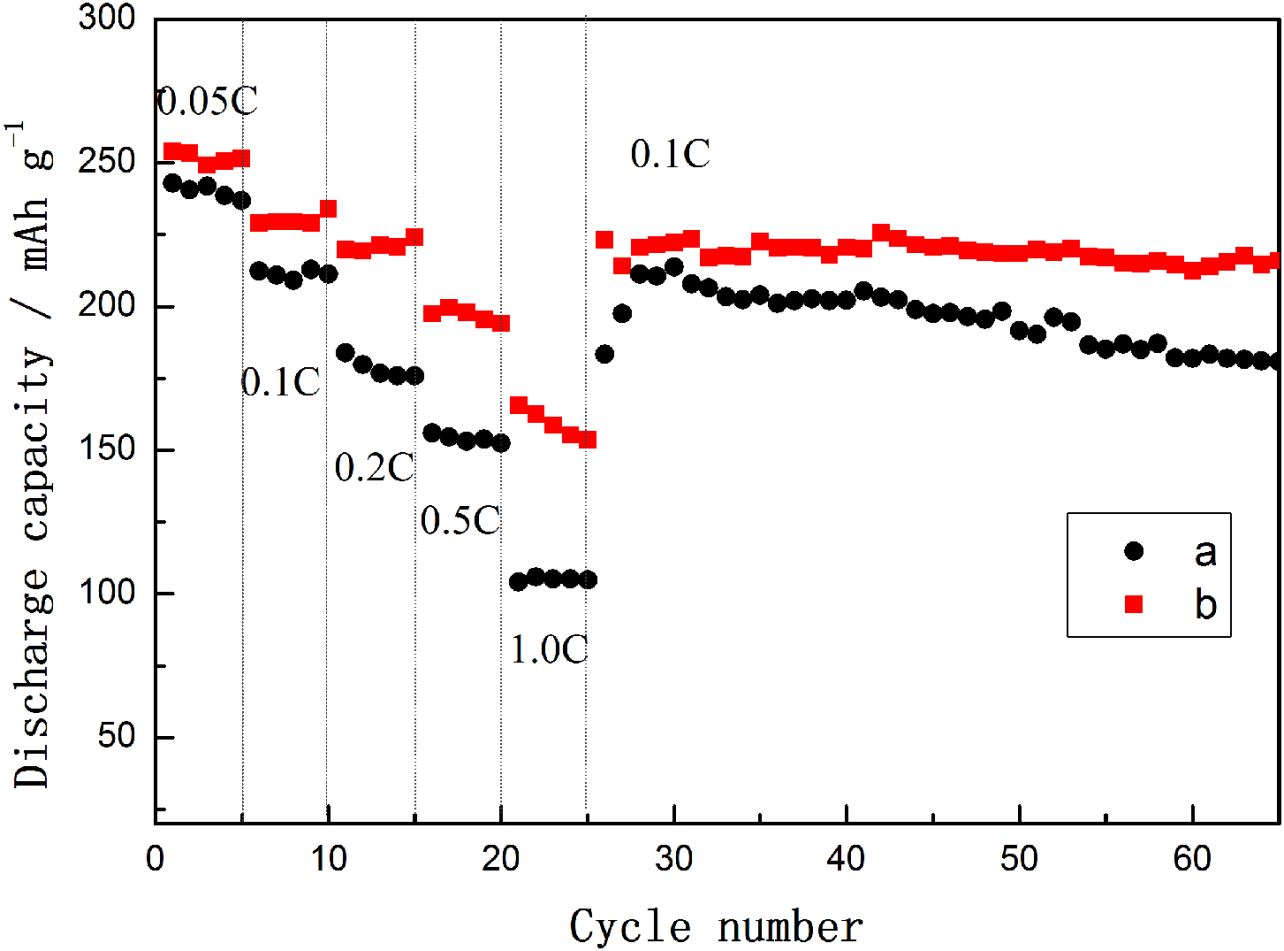
The rate capabilities and cycle performances of (a) TH-LMNO and (b) ILH-LMNO (4.8–2.0 V).

The rate capabilities and cycle performances of TH-LMNO and ILH-LMNO are shown in Figure 7. The current density was increased from 25 mA g^−1^ (0.05 C) to 500 mA g^−1^ (1.0 C) and then switched back to 50 mA g^−1^ (0.1 C). At each rate, the capacity of the ILH-LMNO sample is higher than that of the TH-LMNO sample. These could be attributed to the uniform nanoparticles for the ILH-LMNO sample, which shorten lithium-ion diffusion pathway. When the electrodes are cycled and switched back from 1.0 to 0.1 C, the capacities returned to the initial value, which implied the structural stability of both samples even at a high rate. As displayed in Figure 7, the capacity of the ILH-LMNO sample is also 215.9 mA h g^−1^ after the 65th cycle, with a capacity retention of 94.3% (compared with the first 0.1 C capacity), while the TH-LMNO sample delivered 180.9 mA h g^−1^ with a capacity retention of 85.2%. The obvious capacity degradation observed for the TH-LMNO sample can be attributed to the large particles that possibly limited the lithium-ion diffusion and led to electrochemically inactivated core. The excellent cycling performance of the ILH-LMNO sample is due to the fact that the material belongs to the micro–nanostructure, which is beneficial for the structure stability without apparent kinetic disadvantages.

**Figure 8:**
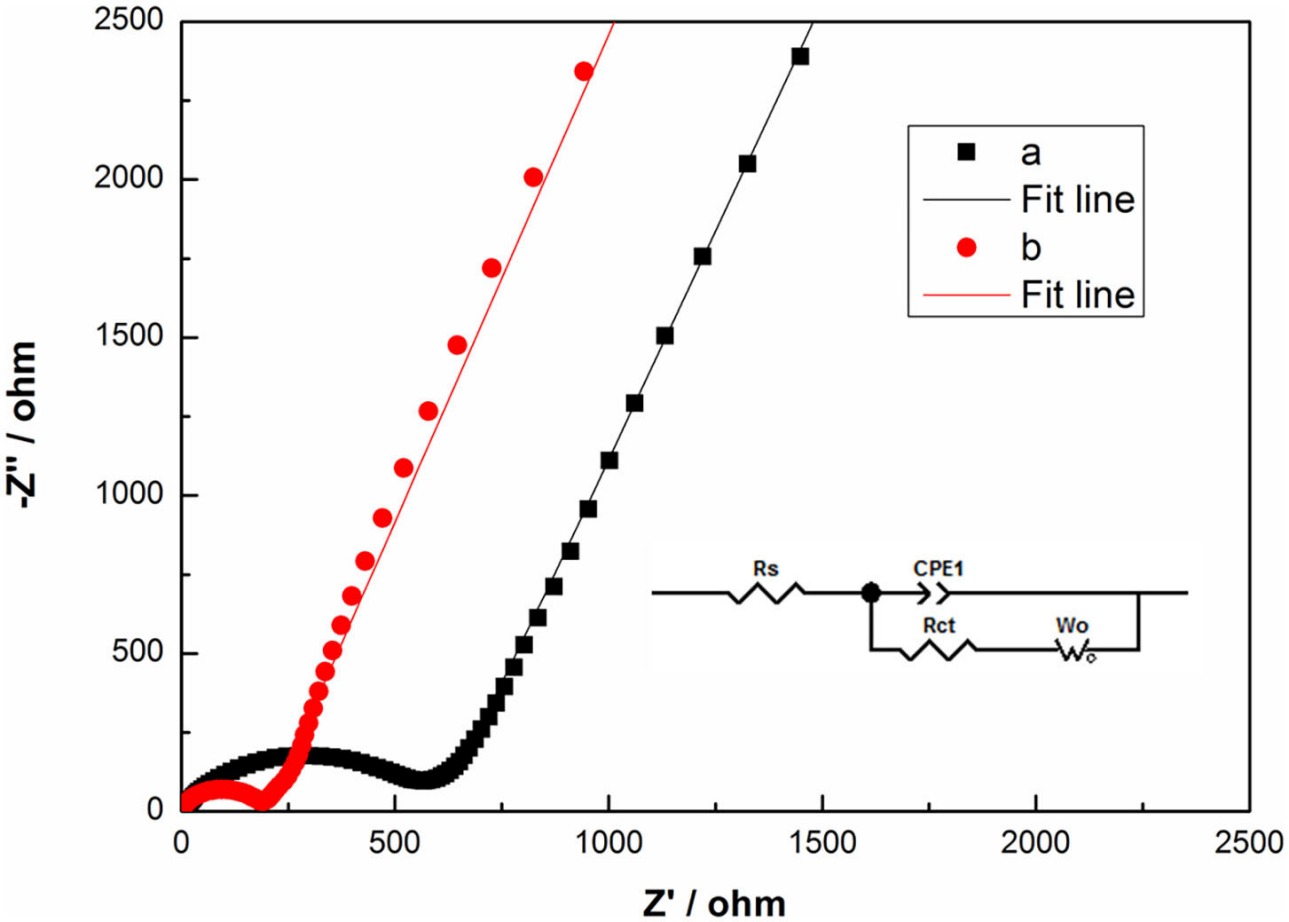
The Nyquist plots of (a) TH-LMNO and (b) ILH-LMNO at the pristine state.

To get insight into the kinetics of the electrode process, EIS of TH-LMNO and ILH-LMNO at the pristine state are measured and shown in Figure 8. These Nyquist plots are well fitted based on the equivalent circuit presented as inset in Figure 8. Rs and Rct refers to the solution resistance and charge-transfer resistance due to the lithium-ion insertion reaction in the electrode/electrolyte interface, respectively, CPE indicates the double-layer capacitance, and Wo represents the Warburg impedance (Wu et al., 2019; Xiang et al., 2019). According to the fitting results, it is found that the Rct value of the ILH-LMNO sample is relatively smaller (155.6 Ω) than that of the TH-LMNO sample (474.6 Ω), which may benefit from the well-dispersed nanoparticles of the ILH-LMNO sample.

## Conclusion

Nano-0.5Li_2_MnO_3_·0.5LiNi_0.5_Mn_0.5_O_2_ cathode materials are synthesized by ionic-liquid-assisted hydrothermal method. XRD results show that the degree of cation mixing and crystalline structure is improved by the ionic liquid during the synthesis. SEM and TEM characterizations suggest that the particles of the materials prepared by the ionic-liquid-assisted hydrothermal method are more uniform and less agglomerated than those prepared by the traditional hydrothermal method. Electrochemical test results indicate that the presence of ionic liquid during the synthesis have a significant effect on the rate capability and cyclability. The 0.5Li_2_MnO_3_·0.5LiNi_0.5_Mn_0.5_O_2_ synthesized by the ionic-liquid-assisted hydrothermal method exhibited a higher initial capacity of 254.1 mA h g^−1^, and the capacity retention after 65 cycles was 94.3%. Besides, the EIS data show that the ionic-liquid-assisted hydrothermal sample have a relatively smaller charge transfer resistance value and thus reduces the diffusion pathways of Li^+^ ions and electrons, which is well consistent with the rate capability test results.

## Data Availability Statement

All datasets generated for this study are included in the article/supplementary material.

## Author Contributions

All authors listed have made a substantial, direct and intellectual contribution to the work, and approved it for publication.

## Conflict of Interest

The authors declare that the research was conducted in the absence of any commercial or financial relationships that could be construed as a potential conflict of interest.
